# Effect of insulin analog initiation therapy on LDL/HDL subfraction profile and HDL associated enzymes in type 2 diabetic patients

**DOI:** 10.1186/1476-511X-12-54

**Published:** 2013-04-24

**Authors:** Ibrahim Aslan, Ertan Kucuksayan, Mutay Aslan

**Affiliations:** 1 Endocrinology Clinic, Antalya Research and Education Hospital, Antalya, Turkey; 2 Department of Medical Biochemistry, Akdeniz University Medical School, Antalya, Turkey

**Keywords:** Diabetes mellitus, Insulin, CETP, LDL, HDL

## Abstract

**Background:**

Insulin treatment can lead to good glycemic control and result in improvement of lipid parameters in type 2 diabetic patients. This study was designed to evaluate the effect of insulin analog initiation therapy on low-density lipoprotein (LDL)/ high-density lipoprotein (HDL) sub-fractions and HDL associated enzymes in type 2 diabetic patients during early phase.

**Methods:**

Twenty four type 2 diabetic patients with glycosylated hemoglobin (HbA1c) levels above 10% despite ongoing combination therapy with sulphonylurea and metformin were selected. Former treatment regimen was continued for the first day followed by substitution of sulphonylurea therapy with different insulin analogs (0.4 U/kg/day) plus metformin. Glycemic profiles were determined over 72 hours by continuous glucose monitoring system (CGMS) and blood samples were obtained from all patients at 24 and 72 hours. Plasma levels of cholesteryl ester transfer protein (CETP), lecithin-cholesterol acyltransferase (LCAT), apolipoprotein B (apoB) and apolipoprotein A-1 (apoA-I) were determined by enzyme-linked immunosorbent assay (ELISA). Measurement of CETP and LCAT activity was performed via fluorometric analysis. Paraoxonase (PON1) enzyme activity was assessed from the rate of enzymatic hydrolysis of phenyl acetate to phenol formation. LDL and HDL subfraction analysis was done by continuous disc polyacrylamide gel electrophoresis.

**Results:**

Mean blood glucose, total cholesterol (TC), triglyceride (TG) and very low-density lipoprotein cholesterol (VLDL-C) levels were significantly decreased while HDL-C levels were significantly increased after insulin treatment. Although LDL-C levels were not significantly different before and after insulin initiation therapy a significant increase in LDL-1 subgroup and a significant reduction in atherogenic LDL-3 and LDL-4 subgroups were observed. Insulin analog initiation therapy caused a significant increase in HDL-large, HDL- intermediate and a significant reduction in HDL-small subfractions. CETP protein level and activity was significantly increased while apoB levels were significantly decreased following insulin analog initiation therapy. No significant difference was found in LCAT mass, LCAT activity, apoA-I and PON-1 arylesterase levels following insulin initiation therapy.

**Conclusion:**

These findings indicate that insulin analog initiation therapy activates lipid metabolism via up-regulating CETP and shows anti-atherogenic effects by increasing HDL-large and decreasing LDL-3 and LDL-4 subfractions in a short time period.

## Introduction

The epidemiological establishment of diabetes as a risk factor for cardiovascular disease is well demonstrated [1]. Even before the development of frank diabetes, insulin resistance causes disturbed lipid transport in plasma [2]. Patients with type 2 diabetes mellitus (T2DM) are frequently associated with low total high-density lipoprotein cholesterol (HDL-C) levels, high levels of small dense LDL and elevated triglyceride (TG) levels [3]. This triad is referred to as the atherogenic lipid profile, which is observed due to insulin resistance [4].

Targeting and treating dyslipidemia improves long-term prognosis in type 2 diabetes [5]. However, despite intervention, these patients remain at increased risk for vascular complications [6], suggesting that other factors may contribute. A part of the increased cardiovascular disease risk in T2DM may be attributed to qualitative changes in lipoprotein subfractions. Although concentrations of Low-density lipoprotein cholesterol (LDL-C) may not be elevated in type 2 diabetes, the dyslipidemia is characterized by an increased proportion of small dense LDL [7] which easily filter into the subendothelial space, are retained by proteoglycans there and are easily oxidized [8]. Indeed, increased small dense LDL particles have been shown to be associated with increased risk of myocardial infarction [9].

Cardiovascular disease risk in T2DM may also be increased by qualitative changes in HDL subfractions with an increased proportion of HDL occurring as smaller, dense HDL [10]. Epidemiological studies showed a predominance of small HDL particles among patients with coronary heart disease as compared with control subjects [11]. HDL also exhibits various anti-atherogenic, anti-oxidant, anti-inflammatory and anti-thrombotic properties [12]. An essential role in these beneficial functions may be played by enzymes and proteins associated with this lipoprotein such as cholesteryl ester transfer protein (CETP), lecithin-cholesterol acyltransferase (LCAT), paraoxonase (PON1) and apoA-I [12].

Although it has been shown that, 2 weeks continuous subcutaneous insulin infusion (CSII) achieved good glycemic control and resulted in an improvement in lipid parameters in newly diagnosed type 2 diabetic patients with fasting glucose levels >200 mg/dl [13], the effect of insulin analog initiation therapy on LDL/HDL subfraction profile and HDL associated enzymes in type 2 diabetic patients has not been established. The aim of this study was to determine the short term effect of insulin analog initiation therapy on LDL/HDL sub-fractions and HDL associated enzymes in type 2 diabetic patients.

## Materials and methods

### Patients

The study group included 24 patients who were admitted to Antalya Research and Education Hospital, Endocrinology Clinic with a diagnosis of T2DM. Patient characteristics and laboratory values are shown in Table 1. The body mass index (BMI) of all patients enrolled in the study was <30 kg/m^2^ and all were non-smokers. None of the patients received antilipidemic agents in the last 3 months before the study. Subjects with apparent history of stroke, coronary heart disease, peripheral artery disease, severe kidney dysfunction, liver disease, thyroid dysfunction, infectious disease, and malignancy were excluded. All subjects enrolled were maintained on a standardized diet before the initiation of the study. HbA1c levels in all patients were above 10% despite ongoing therapy with sulphonylurea and metformin for at least 3 months. Former treatment regimen was continued for the first day followed by substitution of sulphonylurea therapy with different insulin analogs. Patients received either 0.4 U/kg/day lispro mix (50% insulin lispro protamine and 50% insulin lispro) subcutaneously (SC) in three equal doses plus 2000 mg/day oral metformin; 0.4 U/kg/day insulin aspart (30% insulin aspart and 70% protamine insulin aspart) SC in two equal doses plus 2000 mg/day oral metformin; or 0.4 U/kg/day insulin glargine SC in one dose plus 2000 mg/day oral metformin. The given insulin treatments were in accordance with American Association of Clinical Endocrinologists (AACE) Diabetes Mellitus guidelines [14]. All patients gave written informed consent prior to entry. This study was approved by the Institutional Review Board of Antalya Research and Education Hospital and was performed in accordance with the Declaration of Helsinki.

**Table 1:** Patient characteristics and laboratory values

**Variable**	**Mean ± SD**	**n**	**Reference range**
**Age (years)**	52.58 ± 11.43	24	
**BMI (kg/m**^**2**^**)**	25.89 ± 2.26	24	
**BUN (mg/dl)**	12.96 ± 3.50	24	6.00 - 20.00
**Serum Creatinine (mg/dl)**	0.79 ± 0.17	24	0.50 - 1.20
**Microalbumin (mg/24 h)**	24.75 ± 18.15	24	0.00 – 30.00
**ALT (U/L)**	21.71 ± 10.74	24	0.00 – 41.00
**AST (U/L)**	23.88 ± 10.88	24	0.00- 38.00
**TSH (μU/L)**	1.05 ± 0.62	24	0.27 - 4.20

*BMI*, Body mass index; *BUN*, Blood urea nitrogen; *ALT*, Alanine aminotransferase; *AST*, Aspartate aminotransferase; *TSH*, Thyroid stimulating hormone.

### Continuous glucose monitoring

All patients were equipped with continuous glucose monitoring system (CGMS; Medtronic Mini-Med, USA) and were monitored for 72 consecutive hours after admission. A CGMS sensor was inserted into the subcutaneous abdominal fat tissue and calibrated according to the standard Medtronic MiniMed operating guidelines. During CGMS monitoring, blood glucose levels were checked via a glucometer (Accu-Check Go, Roche Co.) 4 times per day and the data was entered into the CGMS. After monitoring for 72 hours, the recorded data were downloaded into a personal computer for analysis of the glucose profile. After downloading the recorded data, mean blood glucose levels were analyzed from the data.

### Laboratory measurements

Blood were obtained from all patients at 24 and 72 hours. HbA1c levels were determined by Abbott ARCHITECT c16000 System (Abbott Diaognostic, Abbott Park Illinois, USA) via immunoturbidimetric method. Total cholesterol (TC), HDL-C and TG were measured on Roche Cobas 8000 Modular Analyser (Basel, Switzerland) via enzymatic colorimetric methods. LDL-C and very low-density lipoprotein cholesterol (VLDL-C) levels were calculated via the Friedewald formula [15]. Blood urea nitrogen (BUN), serum creatinine, alanine aminotransferase (ALT), and aspartate aminotransferase (AST) were measued on Roche Cobas 8000 Modular Analyser via colorimetric methods. Serum thyroid stimulating hormone (TSH) and urine microalbumin was measured on Roche Cobas 8000 Modular Analyser via electrochemiluminescence immunoassay and turbidimetric methods, respectively.

### LDL subfraction analysis

LDL subfraction analysis was performed electrophoretically by use of high-resolution 3% poylacrylamide gel tubes and the Lipoprint LDL System [Quantimetrix, Redondo Beach, California (CA), USA] according to the manufacturer’s instructions [16]. Briefly, 25 μL of sample was mixed with 200 μL of liquid loading gel. The loading gel contained Sudan Black B dye to stain the lipoproteins. The resulting mixture was added to the top of precast 3% polyacrylamide gel tubes. After photopolymerization at room temperature for 35 min, samples were electrophoresed for 1 hour 5 minutes (3 mA/gel tube). Densitometry was performed by Microtek ArtixScan M1 system and data was analyzed by Quantimetrix software (Lipoware-Research version) as previously described [16,17]. In this method, VLDL remains in the origin [retention factor (Rf) = 0.0], whereas HDL migrates at the front (Rf = 1.0). In between, several bands can be detected: Middle (MID) bands C, B, and A, which correspond mainly to intermediate-density lipoprotein (IDL), as well as the LDL bands. LDL1 and LDL2 bands correspond to large, buoyant LDL particles, whereas bands LDL3 and above correspond to sdLDL particles. VLDL and the proportion (%) of the cholesterol mass (mg/dl) of LDL subfractions over the total LDL-C mass were calculated by Quantimetrix software.

### HDL subfraction analysis

Analysis of the apoA-I–containing lipoprotein subfractions was performed using Lipoprint HDL System (Quantimetrix, Redondo Beach, CA, USA) as previously described [18]. Briefly, 25 μl of sample was mixed with 300 μl of Lipoprint Loading Gel and placed upon the upper part of the high-resolution 3% polyacrylamide gel. After 35 min of photopolymerization at room temperature, electrophoresis was performed for 55 min with 3mA for each gel tube. After electrophoresis, VLDL and LDL remained at the origin [Rf = 0.0], whereas albumin migrated at the front (Rf = 1.0). In between, up to 10 bands of HDL can be detected. HDL1 through HDL3 are defined as large HDL; HDL4 through HDL7 are defined as intermediate HDL and HDL8 through HDL10 comprise the small HDL portion. Cholesterol concentration of each HDL subfraction was determined by multiplying the relative area under the curve of each subfraction by the HDL-C concentration of the sample.

### CETP protein and activity measurement

Serum CETP concentrations were analyzed by a commercial enzyme-linked immunosorbent assay (ELISA) test kit [47-CETHU-E01; ALPCO, Salem, New Hampshire (NH), USA] according to the manufacturer’s instructions. Test wells were coated with anti-CETP Monoclonal antibody (Ab). CETP in the sample was captured by the antibody in the 1st incubation. After the 1st incubation and washing to remove all of the unbound material, horseradish peroxidase (HRP)-labeled anti-CETP Monoclonal Ab was added. After the 2nd incubation and subsequent washing, substrate solution was added. Next, stop reagent was added and the intensity of color that develops was measured at 492 nm by a microplate reader. A standard curve of absorbance values of known CETP standards was plotted as a function of the logarithm of CETP standard concentrations (μg/ml) using the GraphPad Prism Software program for windows version 5,03. (GraphPad Software Inc). CETP concentrations in the samples were calculated from their corresponding absorbance values via the standard curve.

Serum CETP activity was measured using an assay kit following the manufacturer’s instruction (BioVision, Mountain View, CA, USA). Briefly, 3 μl of plasma sample (as the source of CETP) was added to the reaction mixture containing a fluorescent self-quenched neutral lipid as the donor molecule and an acceptor molecule. A CETP-mediated transfer of the fluorescent neutral lipid to the acceptor molecule resulted in an increase in fluorescence, which was read in a fluorescence plate reader at excitation 465 nm and emission 535 nm. A standard curve was prepared by using known concentrations (0–100 pmol) of fluorescent neutral lipid standards. CETP activity in the samples was calculated from the corresponding fluorescence values via the standard curve and was expressed as pmol/μl sample/hr.

### LCAT protein and activity measurement

Serum LCAT concentrations were analyzed by a commercial ELISA test kit (47-LCAHU-E01; ALPCO, Salem, NH, USA) according to the manufacturer’s instructions. Test wells were coated with anti-LCAT monoclonal antibody, which binds with LCAT in the sample. After the first incubation and washes to remove all of the unbound material, HRP-labeled anti-LCAT was added. After the second incubation and subsequent washes, the antibody / LCAT / enzyme complex was incubated with a substrate solution and terminated with a stop reagent. The intensity of color that develops was measured at 492 nm by a microplate reader. A standard curve of absorbance values of known LCAT standards was plotted as a function of the logarithm of LCAT standard concentrations (μg/ml) using the GraphPad Prism Software program for windows version 5,03. (GraphPad Software Inc). LCAT concentrations in the samples were calculated from their corresponding absorbance values via the standard curve.

Plasma LCAT activity was assayed using the Calbiochem Fluorometric LCAT assay kit (EMD Bioscience, San Diego, CA, USA) according to the manufacturer’s instructions. This assay is based on the hydrolysis of an artificial LCAT substrate that fluoresces at 470 nm, resulting in a product that fluoresces at 390 nm. Aliquotes of 3 μl of plasma were mixed with 1 μl of fluorescent LCAT substrate and 200 μl of LCAT assay Buffer, followed by incubation for 5 hr at 37°C. The reaction was stopped by adding 300 μl of READ reagent (provided in the kit) to 100 μl of the reaction mixture, followed by fuorometry at 390 and 470 nm. LCAT activity is defined as the change in the ratio of 390/470 nm fluorescence emission intensities.

### Apolipoprotein A1 and B measurements

Serum apoAI levels were determined with the Human ApoAI ELISA kit [no. 3710–1HP-2; Mabtech, Cincinnati, Ohio (OH), USA] according to the manufacturer’s instructions. Samples (diluted 500 000x) were added to ELISA strip plates precoated with apoA1 monoclonal antibody (mAb). Captured apoA1 in the samples were detected by adding a biotinylated mAb followed by streptavidin-HRP. Addition of 3,3’,5,5’ tetramethylbenzidine (TMB) resulted in a colored enzyme substrate solution and the reaction was terminated with a stop reagent. The intensity of color that developed was measured at 450 nm by a microplate reader. A standard curve of absorbance values of known apoA1 standards was plotted as a function of the logarithm of apoA1 standard concentrations (ng/ml) using the GraphPad Prism Software program for windows version 5,03. (GraphPad Software Inc). ApoA1 concentrations in the samples were calculated from their corresponding absorbance values via the standard curve.

Serum apoB levels were determined with the Human ApoB ELISA kit (no. 3715–1HP-2 Mabtech, Cincinnati, OH, USA), according to the manufacturer’s instructions. This kit is specific for detection of apoB100. Samples (diluted 16000 x) were added to ELISA strip plates precoated with apoB100 monoclonal antibody (mAb). Captured apoB100 in the samples were detected by adding a biotinylated mAb followed by streptavidin-HRP. Addition of 3,3’,5,5’ tetramethylbenzidine (TMB) resulted in a colored enzyme substrate solution and the reaction was terminated with a stop reagent. The intensity of color that develops was measured at 450 nm by a microplate reader. A standard curve of absorbance values of known apoB100 standards was plotted as a function of the logarithm of apoB100 standard concentrations (ng/ml) using the GraphPad Prism Software program for windows version 5,03. (GraphPad Software Inc). ApoB100 concentrations in the samples were calculated from their corresponding absorbance values via the standard curve.

### PON1 activity measurement

Serum PON1 activity was determined by a commercial assay kit [ZMC catalogue # 0801199; Zeptometrix Corporation, Buffalo, New York (NY), USA] according to the manufacturer’s instructions. This assay is based on the principle that PON1 catalyzes the cleavage of phenyl acetate, resulting in phenol. The rate of formation of phenol was measured by monitoring the increase in absorbance at 270 nm, at 25°C. One unit of arylesterase activity is equal to 1 μM of phenol formed per minute. The activity is expressed in kU/L, based on the extinction coefficient of phenol of 1310 M^-1^cm^-1^ at 270 nm at pH 8.0 and at 25°C. Blank samples containing water were used to correct for nonenzymatic hydrolysis.

### Statistical analysis

Data were analyzed using Sigma Stat (version 2.03) statistical software for Windows, and a P value < 0.05 was considered statistically significant.

## Results

We performed a two way analysis of variance (ANOVA) to determine whether the three different insulin regimens showed any difference among the reported variables. The subgroup analysis did not uncover any differences between the insulin regimens. Therefore we pooled the groups, and reported the data as before and after insulin treatment.

### Blood glucose and lipid profile

Mean blood glucose, TC, TG and VLDL-C levels were significantly decreased while HDL-C levels were significantly increased after treatment with insulin analogs plus metformin compared to before treatment levels (Table 2). Although not significant, a decrease was also observed in LDL-C levels after treatment with insulin analogs plus metformin (Table 2). Statistical analysis was done by paired t-test.

**Table 2:** Mean blood glucose and lipid profile before and after insulin analog initiation therapy

**Parameter**	**Before treatment** **(mean ± SD)**	**After treatment** **(mean ± SD)**	**n**	**p value**
**Glucose (mg/dl)**	215.46 ± 53.04	177.29 ± 54.48*	24	<0.001
**Total Cholesterol (mg/dl)**	212.50 ± 40.48	203.29 ± 38.86*	24	0.033
**TG (mg/dl)**	257.29 ± 171.87	210.25 ± 130.39*	24	0.001
**VLDL-C (mg/dl)**	51.45 ± 34.37	42.05 ± 26.08*	24	0.001
**LDL-C (mg/dl)**	133.40 ± 35.17	125.62 ± 40.06	23	0.379
**HDL-C (mg/dl)**	33.20 ± 9.28	35.63 ± 10.18*	24	0.003

*TG*, Triglyceride; *VLDL-C*, Very low-density lipoprotein cholesterol; *LDL-C*, Low-density lipoprotein cholesterol; *HDL-C*, High-density lipoprotein cholesterol.

### Changes in LDL subfraction pattern

**Figure 1:**
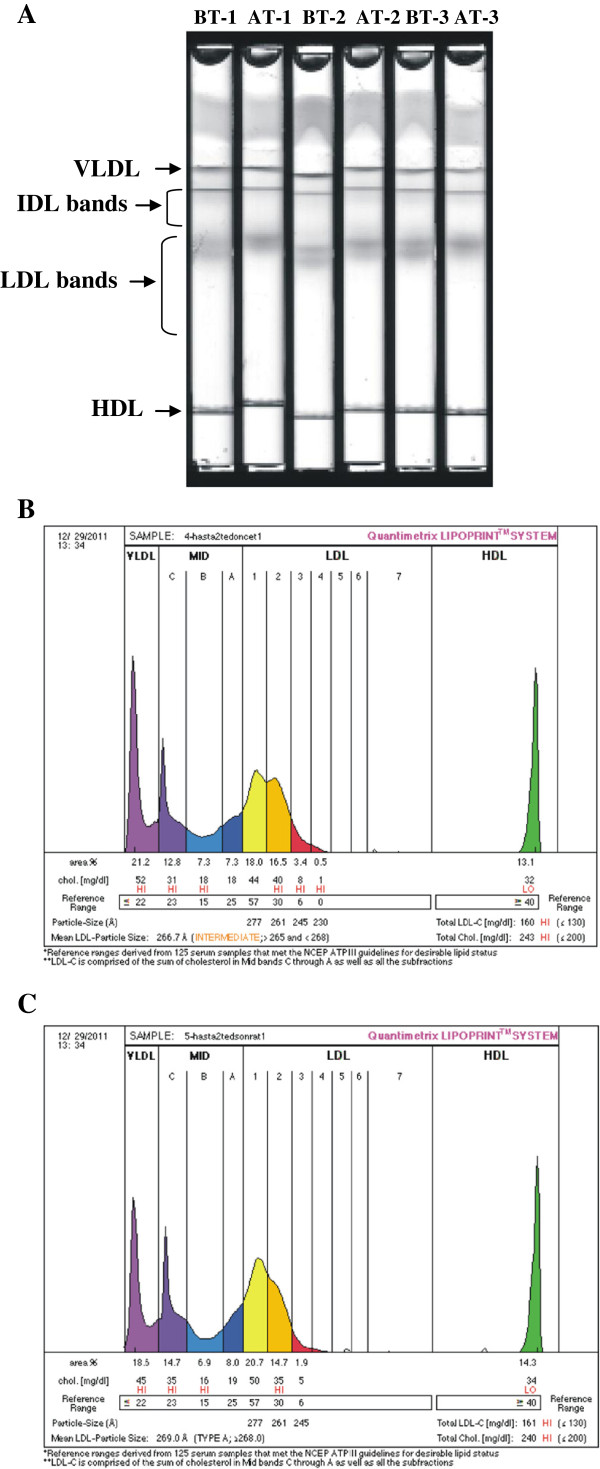
**Electrophoretic separation of lipoproteins on the Quantimetrix Lipoprint Low-Density Lipoprotein System.** (**A**) Image of electrophoretic migration of 6 gel tubes. BT, before treatment; AT, after treatment. (**B**) Demonstrative densitometric scan of a sample before treatment with insulin analogs (**C**) Demonstrative densitometric scan of a sample after treatment with insulin analogs.

Figure 1A shows 6 gel tubes [3 before treatment (BT) and 3 after treatment (AT) with insulin analogs] following completion of electrophoresis. Electrophoretic migration was from the top of the tube to the bottom. Separation was achieved via particle size based on the sieving action of the gel. Chylomicrons remained in the loading gel, VLDL was the slowest migrating band while HDL migrated to a further distance. The LDL particles were separated in the middle part of the gel. Bands corresponding to large, buoyant LDL particles showed clear increase in intensity after treatment with insulin analogs. Figure 1B and 1C are demonstrative densitometric scans of before and after treatment with insulin analogs, respectively. A significant increase in LDL-1 fraction and a significant decrease in LDL-2, LDL-3 and LDL-4 fraction were seen after treatment with insulin analogs (Figure 1B, 1C and Table 3). Statistical analysis was done by paired t-test.

**Table 3:** Low-density lipoprotein subfraction analysis results before and after insulin analog initiation therapy

**Variable**	**Before treatment** **(mean ± SD)**	**After treatment** **(mean ± SD)**	**n**	**p value**
**IDL-C (mg/dl)**	29.63 ± 6.08	28.83 ± 5.48	24	0.481
**IDL-B (mg/dl)**	14.57 ± 3.68	13.99 ± 3.97	24	0.234
**IDL-A (mg/dl)**	14.48 ± 3.99	14.94 ± 4.54	24	0.380
**LDL-1 (mg/dl)**	35.57 ± 10.62	37.96 ± 11.37*	24	0.025
**LDL-2 (mg/dl)**	31.56 ± 10.23	27.68 ± 8.74*	24	0.011
**LDL-3 (mg/dl)**	7.50 ± 6.20	5.33 ± 4.68*	24	0.003
**LDL-4 (mg/dl)**	3.04 ± 2.45	1.70 ± 1.76*	9	0.018

*IDL*, Intermediate-density lipoprotein; *LDL*, Low-density lipoprotein.

### Changes in HDL subfraction pattern

**Figure 2:**
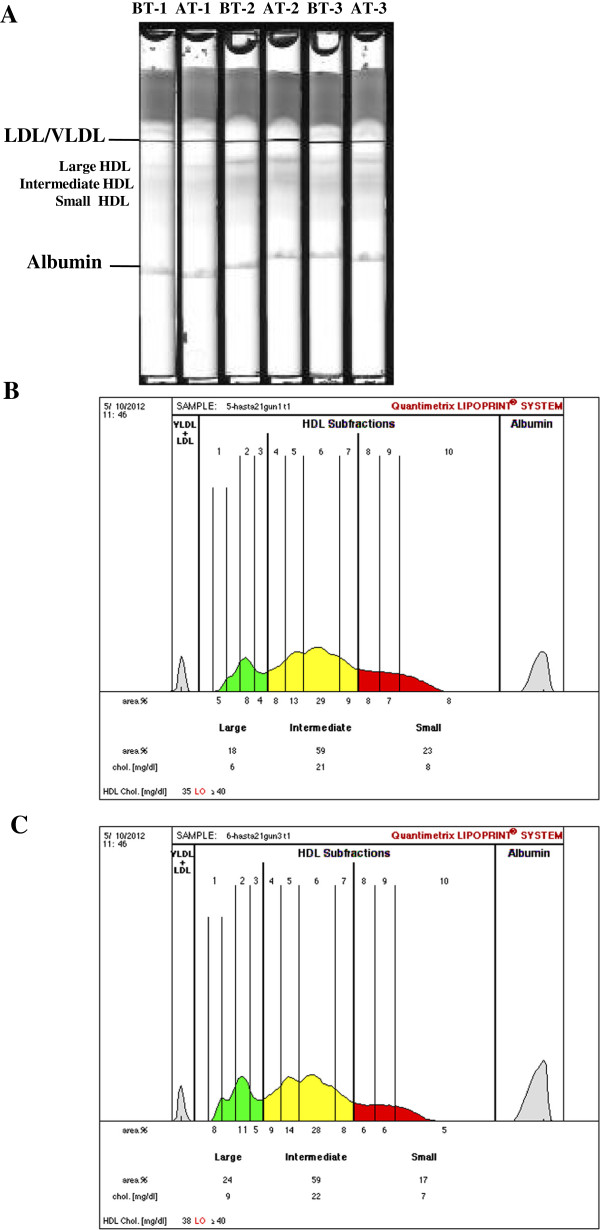
**Electrophoretic separation of lipoproteins on the Quantimetrix Lipoprint High-Density Lipoprotein System.** (**A**) Image of electrophoretic migration of 6 gel tubes. BT, before treatment; AT, after treatment. (**B**) Demonstrative densitometric scan of a sample before treatment with insulin analogs (**C**) Demonstrative densitometric scan of a sample after treatment with insulin analogs.

Figure 2A shows 6 gel tubes [3 before treatment (BT) and 3 after treatment (AT) with insulin analogs] following completion of electrophoresis. Electrophoretic migration was from the top of the tube to the bottom. Separation was achieved via particle size based on the sieving action of the gel. LDL/VLDL was the slowest migrating band while albumin migrated to a further distance. The HDL particles were separated in the middle part of the gel. Bands corresponding to large- and intermediate-HDL particles showed clear increase in intensity after treatment with insulin analogs. Figure 2B and 2C are demonstrative densitometric scans of before treatment and after treatment with insulin analogs, respectively. A significant increase in HDL-large (HDL-1,-2 and-3); HDL-intermediate (HDL-4 and −5) and a significant decrease in HDL-small (HDL-10) fraction were seen after treatment with insulin analogs (Figure 2B, 2C and Table 4). Statistical analysis was done by paired t-test.

**Table 4:** High-density lipoprotein subfraction analysis results before and after insulin analog initiation therapy

**Parameter**	**Before treatment** **(mean ± SD)**	**After treatment** **(mean ± SD)**	**n**	**p value**
**HDL-1 (mg/dl)**	2.88 ± 1.60	3.88 ± 1.93*	24	0.005
**HDL-2 (mg/dl)**	4.43 ± 2.13	5.01 ± 2.32*	24	0.027
**HDL-3 (mg/dl)**	2.46 ± 1.10	3.01 ± 1.16*	24	0.001
**HDL-4 (mg/dl)**	3.79 ± 1.59	4.67 ± 1.39*	24	0.001
**HDL-5 (mg/dl)**	4.69 ± 1.44	5.15 ± 1.24*	24	0.015
**HDL-6 (mg/dl)**	7.72 ±2.19	7.82 ± 2.41	24	0.761
**HDL-7 (mg/dl)**	2.28 ± 0.94	1.99 ±0.82	24	0.204
**HDL-8 (mg/dl)**	1.90 ± 0.76	1.61 ± 0.60	24	0.058
**HDL-9 (mg/dl)**	1.56 ± 0.50	1.36 ± 0.60	24	0.204
**HDL-10 (mg/dl)**	1.42 ± 0.89	0.93 ± 0.58*	24	0.010
**HDL-Large (mg/dl)**	9.57 ± 4.20	12.03 ± 5.25*	24	0.001
**HDL-Inter. (mg/dl)**	18.51 ± 4.99	19.86 ± 5.00*	24	0.018
**HDL-Small (mg/dl)**	4.92 ± 2.02	3.85 ± 1.68*	24	0.012

*HDL*, High-density lipoprotein.

### CETP and LCAT level and activity

**Figure 3:**
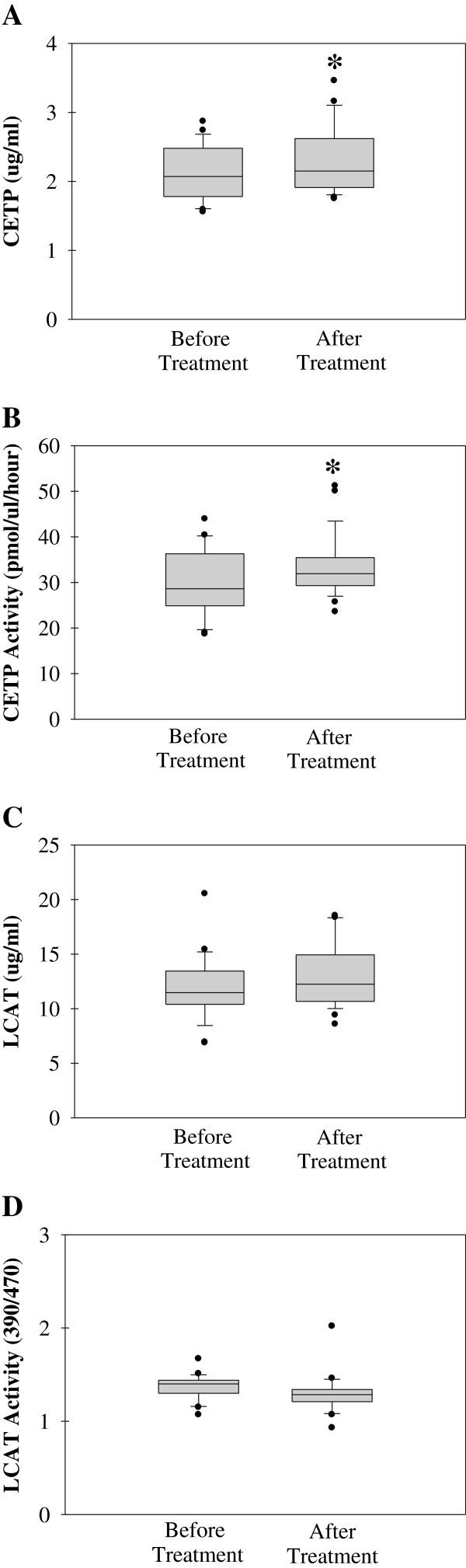
Box plot graph data of (A) plasma cholesteryl ester transfer protein (CETP) levels (B) CETP activity (C) lecithin-cholesterol acyltransferase (LCAT) protein levels (D) LCAT activity.

Box plot graph data of CETP and LCAT protein content and activity are shown in Figure 3. The boundary of the box closest to zero indicates the 25th percentile, the line within the box marks the median, and the boundary of the box farthest from zero indicates the 75th percentile. Whiskers above and below the box indicate the 90th and 10th percentiles.

CETP protein (mean ± SD) measured after treatment with insulin analogs (2.32 ± 0.51 μg/ml) was significantly increased (p < 0.001) compared to before treatment level (2.10 ± 0.39 μg/ml) (Figure 3A). Statistical analysis was done by Wilcoxon Signed Rank Test. Similarly, CETP activity (mean ± SD) measured after treatment with insulin analogs (33.53 ± 6.90 pmol/μl/hr) was significantly increased (p = 0.025) compared to before treatment (30.05 ± 7.15 pmol/μl/hr) (Figure 3B). Statistical analysis was done by paired t-test.

No significant difference was observed in LCAT protein level (mean ± SD) before and after treatment with insulin analogs with measured level of 11.93 ± 2.93 and 12.94 ± 3.05 μg/ml, respectively (Figure 3C). Similarly, no significant difference was observed in LCAT activity (mean ± SD) before and after treatment with insulin analogs with measured ratio of 390/470 nm fluorescence emission intensities of 1.36 ± 0.14 and 1.30 ± 0.22, respectively (Figure 3D).

### Apolipoprotein A1 and B levels

**Figure 4:**
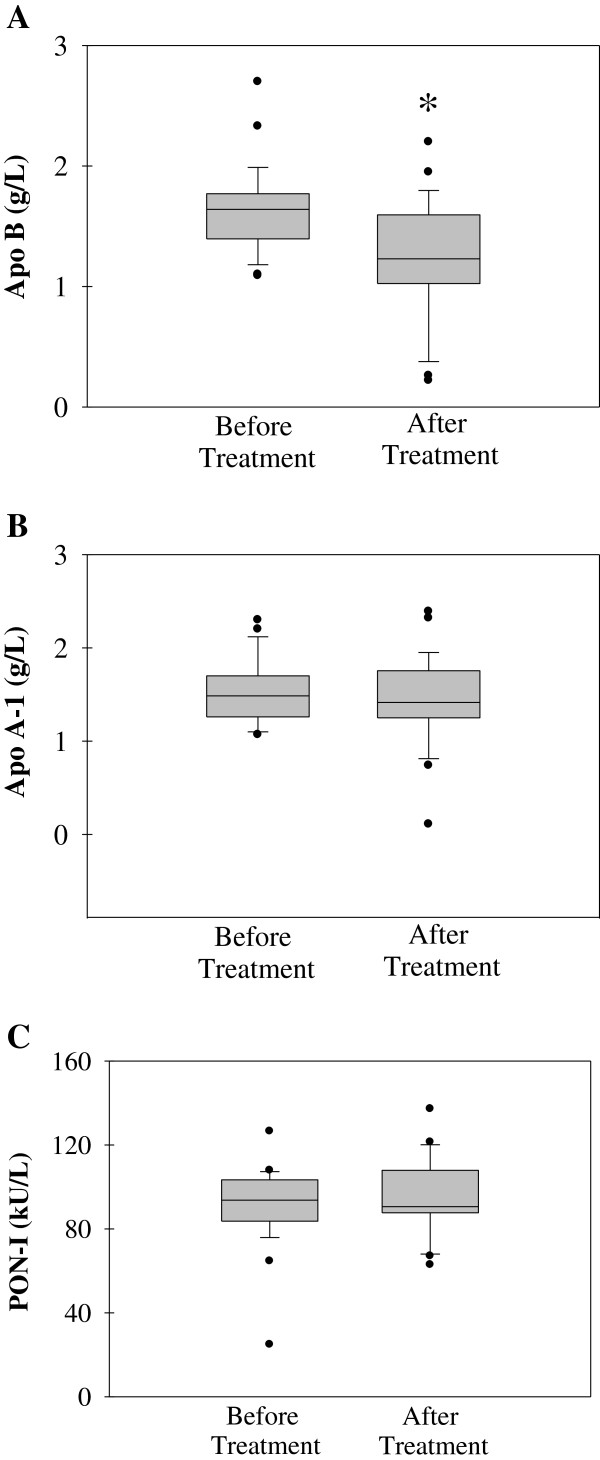
Box plot graph data of (A) Apolipoprotein B 100 protein levels (B) Apolipoprotein A-1 protein levels (C) paraoxonase (PON1) activity.

Apolipoprotein B level (mean ± SD) measured after treatment with insulin analogs (1.25 ± 0.49 g/L) was significantly decreased (p = 0.006) compared to before treatment (1.63 ± 0.37 g/L) (Figure 4A). Statistical analysis was done by paired t-test.

No significant difference was observed in apoA protein level (mean ± SD) before and after treatment with insulin analogs with measured levels of 1.53 ± 0.35 and 1.43 ± 0.49 g/L, respectively (Figure 4B).

### PON1 activity

No significant difference was observed in PON-1 arylesterase activity (mean ± SD) before and after treatment with insulin analogs with measured levels of 91.43 ± 19.33 kU/L and 94.77 ± 18.05 kU/L, respectively (Figure 4C).

## Discussion

Achieving and maintaining glycemic control in type 2 diabetic patients is challenging due to the gradual loss of endogenous insulin secretion and the presence of insulin resistance. The majority of patients are unable to maintain HbA1c targets on a treatment regimen of oral antidiabetic drugs [19]. Thus, addition of insulin therapy is an important step towards achieving long-term glycemic control and reducing the risk of micro- and macrovascular complications [20]. We therefore substituted sulphonylurea therapy with different insulin analogs in patients enrolled in this study.

Modifications of the insulin molecule have resulted in both long-acting insulin analogs such as glargine [21] and rapid-acting insulin analogs such as aspart [22] and lispro [23]. These insulin analogs are reported to have an improved pharmacokinetic/pharmacodynamic profile [24]. Patients enrolled in our study received either lispro mix (50% insulin lispro protamine and 50% insulin lispro) in three equal doses; insulin aspart (30% insulin aspart and 70% protamine insulin aspart) in two equal doses or insulin glargine in one dose. It has been reported that rapid-acting insulin analogs have a faster onset and shorter duration of action than regular human insulin, provide better control of postprandial plasma glucose concentrations [25]. Similarly, Glargine has been shown to provide up to 24-hour glucose control than Neutral protamine Hagedorn (NPH) insulin in patients with type 2 diabetes [26]. A recent guideline from the AACE and the American College of Endocrinology notes that insulin analogs yield better reproducibility and consistency between and within patients [27]. In agreement with reported studies, we have observed that treatment with insulin analog plus metformin resulted in a significant reduction in mean blood glucose levels, as determined by CGMS data.

This investigation demonstrated for the first time that very short-duration insulin therapy (3 days) can significantly alter lipoprotein concentrations in very poorly-controlled Type 2 Diabetic patients who are receiving no lipid therapy. These changes are similar to previous insulin studies but those studies investigated treatment periods from weeks to months and their subjects were not as poorly controlled [13,28]. A more detailed lipoprotein compositional analysis was performed within this study than previous trials. The factors measured within this study would be expected to contribute to changes in lipoprotein composition.

We have observed that TC, TG and VLDL levels were significantly decreased while HDL-C levels were significantly increased after treatment with insulin analogs plus metformin compared to before treatment levels. These findings are in agreement with a previous study that has shown that, 2 weeks CSII achieved good glycemic control and resulted in an improvement in lipid parameters in newly diagnosed type 2 diabetic patients with fasting glucose levels >200 mg/dl [13]. Decreased TC, LDL-C and TG levels and increased HDL-C was observed following short term CSII in the reported study [13]. Similar results were found in another study evaluating the long term (30 weeks) effects of CSII on dyslipidemia in type 2 diabetics without previous history of major cardiovascular disease [28].

We have observed an approximately 17% significant decrease in mean blood glucose levels 48 h after the initiation of insulin analog therapy. According to AACE, insulin therapy should target HbA1c levels of 6.5% or less for most adults [29]. The estimated average glucose level (mg/dl) which corresponds to 7% HbA1c level is reported to be 154 mg/dl (123–185) [30]. Although the achieved reduction in mean blood glucose levels in our study (177.29 ± 54.48 mg/dl) was above the reported target [29,30], we still observed an improvement in lipid parameters as shown in Table 2. Thus, insulin analog initiation therapy has shown beneficial effects on lipoprotein profile in a very short period of time.

We have seen a significant increase in LDL-1 fraction and a significant decrease in LDL-2, LDL-3 and LDL-4 fractions after treatment with insulin analogs. Our findings are in agreement with a previous study that has shown that insulin therapy independently of variations in blood glucose control induces an improvement in LDL subfraction distribution with a shift toward a decrease in small dense LDL in type 2 diabetic patients [31].

We have observed that insulin analog initiation therapy caused a significant increase in HDL-large, HDL- intermediate and a significant reduction in HDL-small subfractions. Our data is in agreement with a previous study which has reported that intensive insulin therapy is associated with increased large buoyant HDL subspecies in type 2 diabetic patients [32]. The observed increase in both LDL-1 and large HDL fractions following insulin therapy may be attributed to the reduction in circulating triglycerides. CETP mediates the transfer of TG from VLDL to HDL and/or LDL in exchange for cholesteryl ester (CE) [33]. It is important to note that CETP does not drive triglycerides or cholesterol esters in one direction or another but is simply a shuttle protein for whatever lipids are available [33]. It is likely that the reduction in circulating triglycerides reduced the exchange of triglycerides in VLDL for cholesterol esters in HDL and LDL, thus, returning them to their normal composition of more large particles and fewer small particle. Our finding of increased CETP activity is in agreement with previous studies which have also shown that insulin treatment increased CETP activity and improved postprandial lipemia [34,35].

We have observed that insulin treatment significantly decreased ApoB levels. ApoB can be degraded in response to decreased VLDL secretion following insulin therapy [36]. When insulin binds to its receptor, the receptor is activated by phosphorylation on tyrosine residues, which phosphorylates insulin receptor substrates 1 (IRS1) and IRS2. The phosphotyrosine residues act as docking sites for the p85 regulatory subunit of phosphatidylinositide 3’ kinase (PI3K) [36]. Insulin suppresses hepatic VLDL production through activation of PI3K [37]. Activated PI3K can be localized to membranes which allows the formation of phosphatidylinositide (3,4,5) triphosphate (PIP3) from phosphatidylinositide (4,5) biphosphate (PIP2). PIP3 is a highly negatively charged phospholipid which may interfere with TG addition into VLDL precursors, thereby reducing the formation of VLDL. VLDL precursors unable to accept lipid droplets are targeted for degradation in lysosomes [38]. ApoB can be degraded in response to decreased VLDL secretion. VLDL overproduction and the loss of insulin suppression of apoB secretion occur in patients with type 2 diabetes [38].

We observed no significant difference in PON-1 arylesterase activity after treatment with insulin analogs. Purified human PON-1 is one of the important components of HDL [39]. PON-1 hydrolyzes several substrates including organophosphates, carboxylic acid esters, lactones and oxidized phospholipids [40]. While over the years enzymatic activity has been named with regard to the substrates required, the same enzyme has been shown to catalyze nearly all arylesterase and paraoxonase activities [41]. The PON-1 activities that have been mostly studied are those towards paraoxon (paraoxonase activity) and phenyl acetate (arylesterase activity) [42]. The PON-1 assay performed in our study was based on the cleavage of phenyl acetate resulting in the formation of phenol.

## Conclusion

In summary, we have observed that insulin analog initiation therapy up-regulates CETP, increases LDL-1 and HDL-large, decreases LDL-3, LDL-4 and small HDL subfractions in a short period of time. The observed changes in lipoprotein profile and CETP occur even though the reduction in mean blood glucose levels are still above the desired target values. Further studies are needed to evaluate the molecular mechanisms by which insulin analogs alter lipoprotein distribution and associated enzymes in reverse cholesterol transport.

## Abbreviations

AACE: American association of clinical endocrinologists; Ab: Antibody; ALT: Alanine aminotransferase; apoA-I: Apolipoprotein A-1; apoB: Apolipoprotein B; AST: Aspartate aminotransferase; AT: After treatment; BMI: Body mass index; BT: Before treatment; BUN: Blood urea nitrogen; CA: California; CE: Cholesteryl ester; CETP: Cholesteryl ester transfer protein; CGMS: Continuous glucose monitoring system; CSII: Continuous subcutaneous insulin infusion; ELISA: Enzyme-linked immunosorbent assay; HbA1c: Glycosylated hemoglobin; HDL: High-density lipoprotein; HDL-C: High-density lipoprotein cholesterol; HRP: Horseradish peroxidase; IDL: Intermediate-density lipoprotein; IRS: Insulin receptor substrates; LCAT: Lecithin-cholesterol acyltransferase; LDL: Low-density lipoprotein; LDL-C: Low-density lipoprotein cholesterol; mAb: Monoclonal antibody; MID: Middle; NH: New Hampshire; NPH: Neutral protamine Hagedorn; NY: New York; OH: Ohio; PI3K: Phosphatidylinositide 3’ kinase; PIP2: Phosphatidylinositide (4,5) biphosphate; PIP3: Phosphatidylinositide (3,4,5) triphosphate; PON1: Paraoxonase; RF: Retention factor; SC: Subcutaneously; SD: Standard deviation; T2DM: Type 2 diabetes mellitus; TC: Total cholesterol; TG: Triglyceride; TMB: Tetramethylbenzidine; TSH: Thyroid stimulating hormone; USA: United States of America; VLDL: Very low-density lipoprotein; VLDL-C: Very low-density lipoprotein cholesterol.

## Competing interest

All authors declare that they have no financial, consulting, and personal relationships with other people or organizations that could influence the presented work.

## Authors’ contributions

IA carried out the clinical studies including enrollment of patients, continuous glucose monitoring, collection of blood samples and contributed in the drafting of the manuscript. EK carried out LDL/HDL subfraction analysis, CETP, LCAT, Apo A1, Apo B and PON1 activity measurements. MA carried out LDL/HDL subfraction analysis, CETP, LCAT, Apo A1, Apo B and PON1 activity measurements and drafted the manuscript. All authors read and approved the final manuscript.
